# Comprehensive analysis of population genetics of *Phoxinus phoxinus ujmonensis* in the Irtysh River: Abiotic and biotic factors

**DOI:** 10.1002/ece3.5320

**Published:** 2019-07-02

**Authors:** Peng Xie, Guang Zhao, Jian‐Gong Niu, Jun Wang, Qiong Zhou, Yan Guo, Xu‐Fa Ma

**Affiliations:** ^1^ College of Fisheries Huazhong Agricultural University Wuhan China; ^2^ Fisheries Research Institute of Xinjiang Uygur Autonomous Region Urumqi China; ^3^ Institute of International Rivers and Eco‐security Yunnan University Kunming China

**Keywords:** cytoplasmic genome, environmental degradation, genetic structure, haplotype diversity, *Phoxinus phoxinus ujmonensis*

## Abstract

As a widely distributed species along the Irtysh River, *Phoxinus phoxinus ujmonensis* (Kaschtschenko, 1899) was used as a model to investigate genetic diversity and population structure as well as the influence of environmental factors on population genetics. In this study, we specifically developed 12 polymorphic microsatellite loci. The analysis of microsatellite and mtDNA markers revealed a high and a moderate genetic diversity across seven populations, respectively. Moderate differentiation was also detected among several populations, indicating the impact of habitat fragmentation and divergence. The absence of isolation by distance implied an extensive gene flow, while the presence of isolation by adaptation implied that these populations might be in the process of adapting to divergent habitats. Correlation analysis showed that abiotic factors like dissolved oxygen, pH, total dissolved solids, and conductivity in water as well as biotic factors like plankton diversity and fish species diversity had impact on genetic diversity and divergence in *P. phoxinus ujmonensis* populations. The results of this study will provide an insight into the effect of environmental factors on genetic diversity and contribute to the study of population genetics of sympatric species.

## 1 INTRODUCTION

In recent decades of research, genetic diversity has been an fundamental and important branch of biodiversity (Campbell, 2003). During the long‐term adaptation and evolution, species could have experienced diversification that resulted from gene flow, selection, genetic drift, and mutation (Rieseberg & Burke, 2001). Genetic diversity is a prerequisite for the survival and evolution of fish since it largely determines the adaptability to environmental changes (Cobben et al., 2012; Shen, Guan, Wang, & Gan, 2016). Fish, especially in nature, often suffers from such environmental changes as environmental degradation, habitat fragmentation, over fishing, and invasion, which affect genetic diversity of fish (Billeter, Billeter, Schmid, & Diemer, 2004; Newbold et al., 2015). These environmental factors could prevent gene flow among populations, accelerate selection, genetic drift, and mutation and subsequently promote the formation of significant genetic structure (Nosil, 2010; Wright, 1990). However, genetic structure could reflect demographic histories, especially the level of inbreeding and migration among populations (De Barba et al., 2010; Mckinnon & Rundle, 2002). In addition, fish from different geographic populations may also exhibit the patterns of isolation by distance (IBD) or isolation by adaptation (IBA; Hélène & Luca, 2011; Luisa, Joost, Ine, Joachim, & Luc, 2013; Mendez, Rosenbaum, Subramaniam, Yackulic, & Bordino, 2010; Polato, Concepcion, Toonen, & Baums, 2010; Ruiz‐Gonzalez, Cushman, Madeira, Randi, & Gomez‐Moliner, 2015). Thus, it is of great importance to understand the genetic diversity and structure of fish species so as to provide information for species biodiversity and conversation.

**Figure 1:**
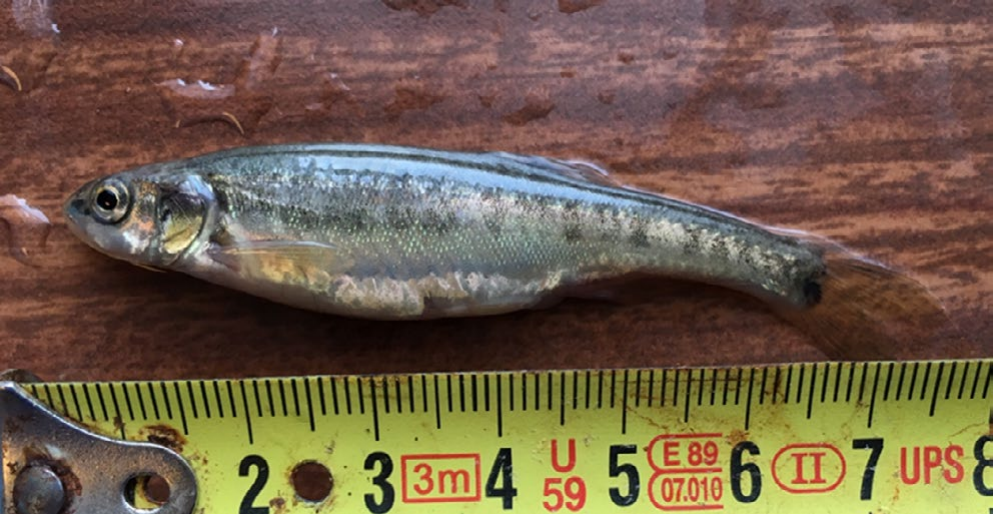
Photograph of an individual of *Phoxinus phoxinus ujmonensis* captured from KYET population in 2015

The Irtysh River is one of the biggest cross‐border rivers in the northwest of China, flowing 546 km within China through Kazakhstan and Russia, and eventually into the Arctic Ocean. In China, there are about 23 native species in this river basin. However, fish populations have declined sharply in recent decades (Guo, Zhang, & Li, 2003; Huo et al., 2010). Hence, the situation of fish populations and structure has attracted wide interest and concerns. Notably, *Phoxinus phoxinus ujmonensis*, a small cold freshwater fish (Figure 1), is the only one species that is widely distributed throughout almost the whole Irtysh River basin (especially in China). In this watershed, both abiotic and biotic factors differ greatly along upper stream, lower stream, and tributaries, such as water temperature, conductivity, and total dissolved solids. Moreover, there exist several water conservancy facilities throughout this river basin. These factors might have induced the fragmentation and divergence of the habitats of fish，which may also become barriers to gene flow. Our previous investigations of feeding habits of predatory fish like *Lota lota* in Irtysh River indicated that *P*. *phoxinus ujmonensis* was prey to almost all these fish in this river. Obviously, *P*. *phoxinus ujmonensis* possesses a significant niche in the food web within this river. Thus, it could act as a good bio‐model for the study of the population diversity and structure, as well as the effect of environmental factors on diversity within the Irtysh River. However, little research on population genetics of fish in the cold northwest areas of China has been available.

This study conducted an analysis of the population genetics of *P*. *phoxinus ujmonensis* with specifically developed markers and correlation analysis of genetic indexes and abiotic or biotic factors so as to deepen our understanding of the relationship between environmental factors and genetic diversity and structure.

## 2 MATERIALS AND METHODS

### 2.1 Ethics statement of sampling and DNA extraction

**Figure 2:**
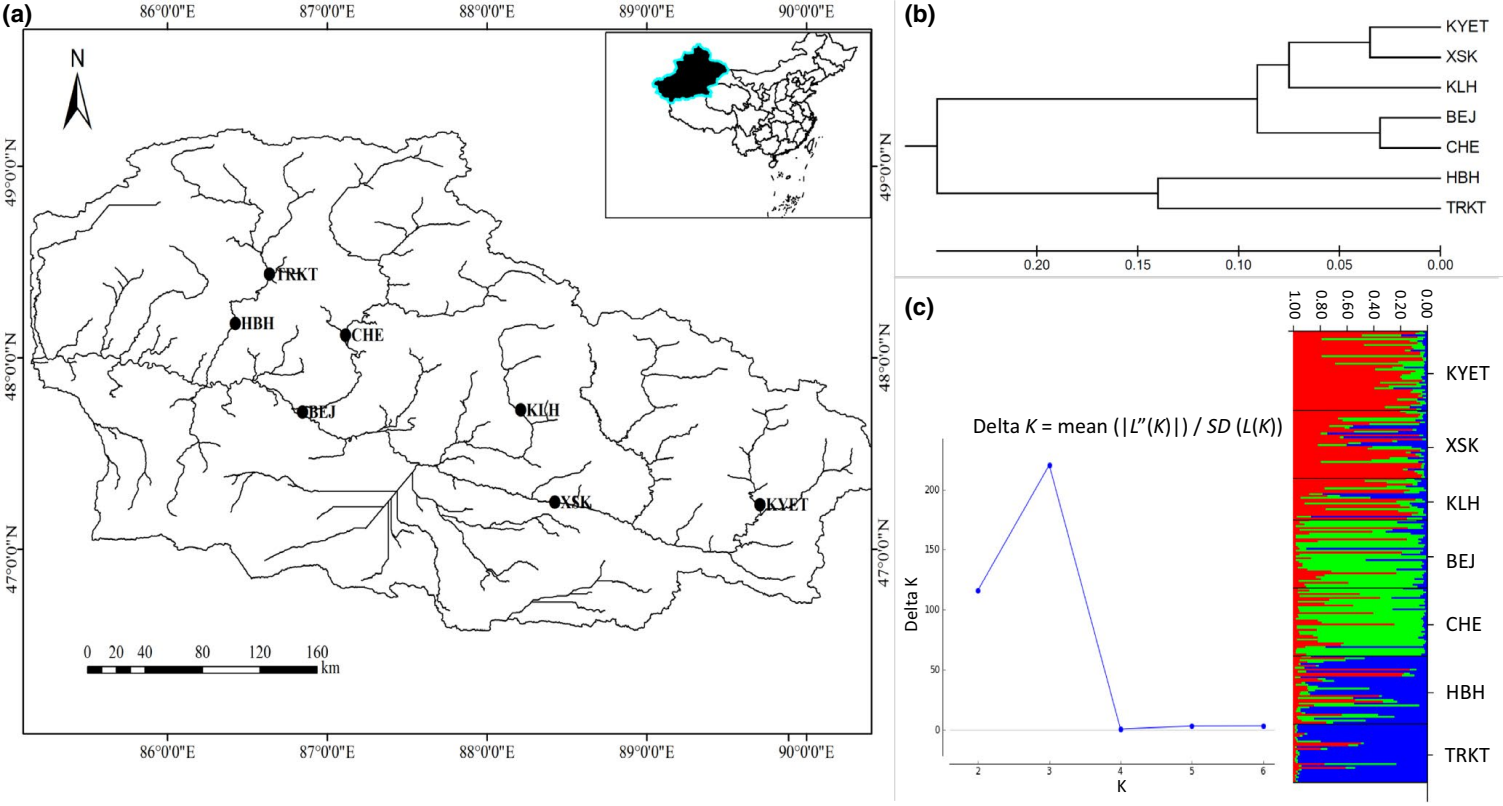
Locations of *Phoxinus phoxinus ujmonensis* populations sampled, map was constructed in ArcGIS 10.4.1. (a); unweighted pair‐group method with arithmetic means (UPGMA) dendrogram based on *F*_st_p_ values from microsatellite data, constructed in Mega 6.0 (b); model choice criterion ln*P*(*D*) of each *K* value for the STRUCTURE analysis and STRUCTURE analysis of microsatellite loci variation among seven populations, calculated and visualized in STRUCTURE 2.3.4. (c). KYET: *n* = 42, XSK: *n* = 36, KLH: *n* = 22, BEJ: *n* = 36, CHE: *n* = 36, HBH: *n* = 36, TRKT: *n* = 31

We confirmed that the sampling areas were not privately owned or preserved ones. We only sampled the individuals related to this study, involving no endangered or protected species. It was ensured that all the experiments and fish individuals involved were in accordance with the “Guidelines for Experimental Animals” of the Ministry of Science and Technology (Beijing, China; No. [2006]398, 30 September 2006). We kept the damage to the fish population to the minimum, especially in the breeding season. All the sampling work was supported by the local institution Fisheries Research Institute of Xinjiang Uygur Autonomous Region, Urumqi 830000, China. From July 2013 to September 2015, we finished the 10 times sampling work and collected a total of 239 individuals from seven locations (Figure 2a; Table ). Fins were cut off the fish and immediately stored into the ethanol. Total genomic DNA samples were extracted from individuals according to the standard Proteinase‐K/phenol‐chloroform protocol (Russell & Sambrook, 2001). The concentration of all the DNA samples was detected by the Micro‐plate Spectrophotometer (Epoch; BioTek) and diluted to 100 ng/µl.

### 2.2 Isolation and characterization of microsatellite loci

Genomic libraries of 300–900 bp fragments were constructed according to the method described as Bei et al. (2012). The microsatellite library was subsequently constructed by magnetic bead enrichment method (Cui‐Yun, 2005; Li, Gruber, Jessee, & Lin, 1998; Zane, Bargelloni, & Patarnello, 2010). Positive colonies in the plates of *Escherichia coli* DH5α cells were screened by polymerase chain reaction (PCR) using universal M13 primers. Plasmid DNAs extracted from positive colonies were sequenced, and sequencing results were analyzed with the SSRHunter v 1.3.0 (Li & Wan, 2005) to locate the microsatellite loci. Afterward, the primer pairs of microsatellites were designed using Primer premier 5.0 software.

In order to determine the characteristics of primer pairs, we tested these primer pairs on 36 *P. phoxinus ujmonensis* individuals collected from Haba River (HBH population). PCR amplifications were performed in a 10 µl of solution containing 50–100 ng genomic DNA, 2 × Es Taq MasterMix (Es Taq DNA Polymerase, 2 × Es Taq PCR Buffer, 3 mM MgCl_2_, 400 µM dNTP mix, CWBIO), and 5 µM of each primer on a Applied Biosystems (ABI). Thermal cycling parameters were as follows: initial denaturation at 94℃ for 3 min, followed by 35 cycles of denaturation at 94℃ for 30 s, specific annealing at different temperatures (Table 1) for 35 s, extension at 72℃ for 40 s, and final extension at 72℃ for 8 min. All PCR products were resolved on 1.5% agarose gels to screen out primer pairs that could exhibit consistent amplification of polymorphic loci, and then, these primer pairs were further screened by polyacrylamide gel electrophoresis (PAGE). Subsequently, the images of PAGE were analyzed by Quantity One v 4.6.2 (Yáñez et al., 2008). Allele size ranges, the number of alleles per locus (*N*_a_), heterozygosity, and polymorphic information content (PIC) were computed using Popgene v 1.32 and PIC Calculator v 0.6 (Kemp, 2002).

**Table 1:** Main information of 12 microsatellite loci and two mitochondrial genes for *Phoxinus phoxinus ujmonensis*

Loci	Core sequence	Forward primer	Reverse primer	Dye	*T*_a_ (℃)	*L*_a_ (bp)	Accession no.
ppu17	(GT)7…(GT)7…(GT)6	CGATGAGACCGTTACCAAA	ATGAACAAGAACCGACCC	FAM	56	300	KJ996067
ppu22	(TG)19	AATGAGCGTGGTAATGAA	ATAGGTCTACTTTGTGGC	FAM	55	104	KM006334
ppu23	(TG)18	ATGTAATAAGCATAGGCGTC	CCGAAAAACCCTCAGGAACT	FAM	54	136	KM006335
ppu28	(CA)17…(CA)7	CTCACACTCAAACTCTTCTCC	GCTTCACCTGTTGATAGTCT	HEX	54	155	KM106213
ppu29	(TG)8…(GT)22	CCTGAACACCAGCGTAAG	CAGCGGGAAACTGAGCAT	TAMRA	56	244	KM006336
ppu33	(CA)10	AGACTCGCTCCATCATTCA	GCTCTTCCCGTTCTCCTC	FAM	55	142	KM006337
ppu40	(CA)11…(AC)8…(AC)10	GAGAATCCAGAGCATCT	TAAACAACATTCCGTCT	HEX	55	177	KM006338
ppu44	(GT)13	TTGACGGACATCTGGACAC	CATGCTGGGAAATGGTTTA	TAMRA	54	263	KM006339
ppu45	(CA)13…(CA)11…(AC)5	CAGAGCGTCCTACATC	GGTCCCCATCCAACAT	TAMRA	55	243	KM006340
ppu47	(CA)36	GAATAGAGCCGCAGCACAT	TCCTTTACAATCAGTCCCT	TAMRA	56	243	KM006341
ppu49	(GT)35	TTTCTGCGTCAGTTTGTG	GACCTGGCTGTCTGCTAA	HEX	55	202	KM006342
ppu51	(GT)42	ATGCCCTTGCTGAACGACT	CCTTCTCCCGCTCACGATA	HEX	55	214	KM006343
*Cox I*	Xie et al. (2016	GCTTTTGACTTCTACCCC	GAACTTGGACGAATGCTG	‐	55	1,143	KJ000673 a
*Cox II*		TTCAAGCCAGTCACATAAC	TAGAGGTGGTCGGTAATC	‐	55	691	

Note

*L*_a_, length of the amplification; *T*_a_, annealing temperature.

a This accession number was connected to the complete mitochondrial genome of *P. phoxinus ujmonensis* that contained detailed location and full length of these two genes.

### 2.3 Microsatellite loci genotyping and statistical analysis

In order to ensure the stability and reliability of the results, we selected microsatellite loci of good quality to analyze the genetic diversity of the seven *P*. *phoxinus ujmonensis* populations. Forward primers were 5′ modified with a fluorescent dye (FAM, HEX, or TAMRA, Table 1). PCR amplifications were carried out as described above. All PCR products were genotyped on an ABI PRISM 3730 genetic analyzer (Applied Biosystems) with a ROX GS400 size standard. The genotype results were analyzed by GeneMarker v 1.51 (Holland & Parson, 2011). Afterward, the data file obtained from GeneMarker was converted into specific formats by CONVERT (Glaubitz, 2004) to evaluate the population genetics indexes with several softwares. The number of alleles (*N*_a_), number of effective alleles (*N*_e_), expected heterozygosity (*H*_e_), and observed heterozygosity (*H*_o_) were calculated for each locus and each population with PopGene v 1.32 (Yeh, Yang, & Boyle, 1999). Nei's unbiased genetic distance (*N*_d_), Shannon's information index (*S_i_*), and deviations from Hardy–Weinberg equilibrium (HWE) were computed through both Popgene and GenAlEx 6.5 (Peakall & Smouse, 2006). The analysis of population specific fixation index (*F*_st_), pairwise genetic differentiation coefficient (*F*_st_p_), and the hierarchical analysis of molecular variance (AMOVA) were performed using Arlequin v 3.5.1.2 (Excoffier & Lischer, 2010). Based on pairwise *F*_st_p_ values, the unweighted pair‐group method with arithmetic means (UPGMA) dendrogram was constructed with Mega 6.0 (Tamura, Stecher, Peterson, Filipski, & Kumar, 2013).

We performed the principal coordinate analysis (PCoA) with GenAlEx 6.5 to visualize the patterns of genetic relationships between individuals and populations, basing on pairwise Euclidean distances. Admixture analysis was conducted in STRUCTURE 2.3.4 (Evanno, Regnaut, & Goudet, 2005) by a Bayesian clustering method to determine most possible number of genetic clusters, regardless of sampling population information. Analyses were performed by a burn‐in of 50,000, followed by 500,000 MCMC repetitions with main parameters set as admixture model and correlated allele frequencies. Each cluster set (*K* = 1–7) was run at iteration of 10. Subsequently, the delta‐*K* (Δ*K*) metric of these cluster sets was uploaded to the web interface STRUCTURE HARVESTER (http://taylor0.biology.ucla.edu/structureHarvester/; Earl, 2012) to calculate the statistically most supported number of clusters.

### 2.4 Mitochondrial DNA sequence amplification and statistical analysis

In our previous study, we had sequenced and characterized the complete mitochondrial genome of *P. phoxinus ujmonensis* (Xie et al., 2016). Thus, we located the *Cox I* gene and *Cox II* gene and designed primer pairs with Primer Premier 5.0 (Table 1). PCR amplifications were conducted in the same reaction system described above. Thermal cycling parameters were as follows: initial denaturation at 94℃ for 3 min, followed by 35 cycles of denaturation at 94℃ for 30 s, specific annealing at 55℃ for 35 s, extension at 72℃ for 40 s, and final extension at 72℃ for 8 min. Raw data of *Cox I* + *Cox II* were edited with EditSeq software in DNASTAR package v 7.1 (Burland, 2000) and aligned with Mega 6.0, and the concatenate sequences of *Cox I* + *Cox II* were subjected to the same edition and alignment. Haplotype diversity (*H*_d_) values and nucleotide diversity (*P_i_*) values were calculated, and neutrality test of each population was conducted using DnaSP v 5.1 (Librado & Rozas, 2009). Based on these haplotypes, the median‐joining networks were constructed in Popart v 1.7 (Bandelt, Forster, & Röhl, 1999; Leigh & Bryant, 2015). Genetic distances (*G*_d_) of these seven groups were calculated with Mega 6.0. To better understand the genetic distance and structure of these populations, we constructed the phylogenetic tree of all the 239 individuals based on the *Cox I* + *Cox II* dataset in Mega 6.0. We determined the best‐fitting nucleotide substitution model which produced the lowest Bayesian information criterion (BIC) score using Mega 6.0. (Tamura et al., 2013).

### 2.5 Correlation analysis of genetic data and environmental data

To investigate the relationship between population genetic characteristics and environmental factors, we collected some abiotic data such as water temperature (*T*), dissolved oxygen (DO), and conductivity (Con) from 2012 to 2017, as well as computed biotic indexes, such as plankton diversity indexes (Mar: Margalef index, Sha: Shannon–Wiener's index, Sim: Simpson's diversity index, and Pie: Pielou index) and Shannon–Wiener's diversity indexes of fish species (*S*_f_, for the three regions described below; Table). We conducted the principal component analysis (PCA) with the “princomp” function in R 3.5.3. We conducted Mantel tests via the “vegan” package in R 3.5.3 to determine whether there exist IBD and IBA patterns (Hélène & Luca, 2011; Oksanen et al., 2013). We subsequently analyzed and visualized the correlations between genetic diversity indexes and environmental factors in R 3.5.3.

## 3 RESULTS

### 3.1 Characteristics of microsatellite loci

We designed a total of 54 primer pairs, based on 226 clones. Among these 54 microsatellite loci, 19 loci were perfect type, seven were interrupted type, and the others were compound type (Mathithumilan et al., 2013). After PAGE test, 12 microsatellite loci turned out to be polymorphic and of good quality, compared with the loci screened from cross‐species amplification in *Phoxinus phoxinus* (L.) (Holmen, Vøllestad, Jakobsen, & Primmer, 2009). Core information of these loci was submitted to GenBank (Table 1).

### 3.2 Genetic diversity

#### 3.2.1 Microsatellite dataset

All the 239 individuals from seven populations were genotyped in terms of the 12 microsatellite loci. However, the ppu23 did not consistently exhibit the expected amplifications across some populations, and significant deviations from Hardy–Weinberg equilibrium (HWE) were observed in ppu17, ppu47, ppu49, and ppu51 among several populations. Thus, we abandoned the data of the above five loci to ensure the reliability of genetic results (Planes & Fauvelot, 2002). For the seven polymorphic loci employed, the *N*_a_, *N*_e_, *H*_o_, *H*_e_, and *S_i_* values of each population were calculated (Table 2). The highest *H*_o_, *H*_e_, and *S_i_* values of the HBH population suggested that it maintained the highest genetic diversity.

**Table 2:** Population genetic diversity of *Phoxinus phoxinus ujmonensis* across seven polymorphic microsatellite loci

	KYET (*n* = 42)			XSK (*n* = 36)			KLH (*n* = 22)			BEJ (*n* = 36)			CHE (*n* = 36)			HBH (*n* = 36)			TRKT (*n* = 31)		
	*N*_a_	*N*_e_	HWE	*N*_a_	*N*_e_	HWE	*N*_a_	*N*_e_	HWE	*N*_a_	*N*_e_	HWE	*N*_a_	*N*_e_	HWE	*N*_a_	*N*_e_	HWE	*N*_a_	*N*_e_	HWE
ppu22	8	5.61	0.135	9	5.96	0.140	8	4.89	0.079	9	6.86	0.163	9	6.51	0.101	9	5.29	0.381	8	4.02	0.326
ppu28	16	6.81	1.000	14	5.84	0.644	15	10.52	0.865	17	9.09	0.696	13	7.83	0.113	17	10.09	0.590	11	5.65	0.196
ppu29	16	5.76	1.000	15	6.58	0.061	13	9.78	0.723	14	8.10	0.342	13	6.35	0.181	15	9.89	0.207	13	5.41	0.225
ppu33	9	6.29	0.197	10	5.61	0.237	8	5.47	0.065	12	8.25	0.095	9	4.88	*0.018*	11	8.00	0.256	9	5.72	0.062
ppu40	9	4.08	0.755	8	4.26	0.586	11	7.22	0.301	9	6.16	0.758	9	4.58	0.808	9	5.51	*0.034*	7	4.05	0.414
ppu44	14	6.26	0.124	11	6.17	0.051	9	4.44	0.254	10	5.13	0.565	11	5.67	0.605	11	7.36	0.968	10	7.20	0.213
ppu45	12	2.76	0.198	8	2.62	0.090	9	3.90	0.083	7	3.04	0.548	7	2.69	0.349	9	5.51	0.142	9	4.32	0.729
Mean	12.00	5.36		10.71	5.29		10.43	6.60		11.14	6.66		10.14	5.50		11.57	7.38		9.57	5.20	

	*H*_o_	*H*_e_	*S_i_*	*H*_o_	*H*_e_	*S_i_*	*H*_o_	*H*_e_	*S_i_*	*H*_o_	*H*_e_	*S_i_*	*H*_o_	*H*_e_	*S_i_*	*H*_o_	*H*_e_	*S_i_*	*H*_o_	*H*_e_	*S_i_*
ppu22	0.857	0.832	1.850	0.861	0.844	1.932	0.909	0.814	1.813	0.861	0.866	2.027	0.806	0.858	1.987	0.917	0.822	1.881	0.839	0.764	1.615
ppu28	0.976	0.864	2.283	1.000	0.840	2.105	0.864	0.926	2.512	0.861	0.903	2.445	0.944	0.885	2.221	0.889	0.914	2.505	0.903	0.837	1.992
ppu29	0.976	0.836	2.140	0.833	0.860	2.222	0.773	0.919	2.397	0.861	0.889	2.286	0.833	0.855	2.104	0.833	0.912	2.454	0.839	0.829	2.052
ppu33	0.738	0.851	1.979	0.778	0.833	1.950	0.727	0.836	1.843	0.806	0.891	2.269	0.806	0.806	1.834	0.806	0.887	2.212	0.807	0.839	1.924
ppu40	0.810	0.764	1.715	0.833	0.776	1.703	0.818	0.882	2.151	0.833	0.849	1.952	0.750	0.793	1.806	0.778	0.830	1.870	0.710	0.766	1.542
ppu44	0.667	0.850	2.183	0.667	0.850	2.064	0.636	0.793	1.814	0.667	0.817	1.919	0.750	0.835	2.014	0.833	0.876	2.133	0.839	0.875	2.063
ppu45	0.595	0.645	1.590	0.556	0.627	1.339	0.636	0.761	1.731	0.611	0.680	1.439	0.500	0.637	1.330	0.694	0.830	1.887	0.677	0.781	1.724
Mean	0.803	0.806	1.963	0.790	0.804	1.902	0.766	0.847	2.037	0.786	0.842	2.048	0.770	0.810	1.900	0.821	0.867	2.135	0.802	0.813	1.845

Abbreviations:*N*_a_, number of alleles; *N*_e_, number of effective alleles; *H*_e_, expected heterozygosity; *H*_o_, observed heterozygosity; HWE, *p* values of Hardy–Weinberg equilibrium test; *S_i_*, Shannon's information index.

#### 3.2.2 Mitochondrial dataset

We analyzed the 1,143 bp mtDNA fragments of *Cox I* gene with full length being 1,551 bp, the 691 bp mtDNA fragments of *Cox II* gene at full length (Table 1), and the 1,834 bp concatenated fragments of *Cox I + Cox II*. In total, 59, 29, and 82 haplotypes were detected from *Cox I*, *Cox II*, and *Cox I + Cox II* dataset in all the 239 individuals, respectively (Table 3). These results presented moderate haplotype diversity and low nucleotide diversity.

**Table 3:** Number of haplotypes (*N*), haplotype diversity (*H*_d_), and nucleotide diversity (*P_i_*) revealed by mtDNA markers

	*Cox Ⅰ*			*Cox Ⅱ*			*Cox Ⅰ* + *Cox Ⅱ*		
	*N*_h_	*H*_d_	*P_i_*	*N*_h_	*H*_d_	*P_i_*	*N*_h_	*H*_d_	*P_i_*
KYET	7	0.587	0.00093	7	0.561	0.00164	10	0.746	0.00120
XSK	19	0.852	0.00202	10	0.671	0.00155	23	0.913	0.00184
KLH	6	0.476	0.00055	6	0.411	0.00079	11	0.714	0.00064
BEJ	11	0.594	0.00072	8	0.440	0.00101	16	0.752	0.00083
CHE	11	0.522	0.00058	2	0.056	0.00008	12	0.560	0.00039
HBH	12	0.784	0.00152	7	0.354	0.00064	16	0.875	0.00119
TRKT	8	0.576	0.00104	3	0.546	0.00083	9	0.799	0.0009666
All‐pops	59	0.663	0.00115	29	0.486	0.00107	82	0.802	0.00112

### 3.3 Population structure

The *N*_d_ values of the seven populations ranged from 0.0928 (KYET and XSK) to 0.7957 (TRKT and CHE), while the *F*_st_p_ values varied from 0.01319 (BEJ and CHE) to 0.11049 (TRKT and CHE; Table 4). Notably, moderate differentiation was detected between TRKT population and the rest five populations except HBH population, which might be attributed to the fact that both HBH and TRKT population were located in the same tributary.

**Table 4:** Nei's unbiased genetic distance (*N*_d_, below diagonal) and genetic differentiation coefficient (*F*_st_p_, above diagonal) revealed from microsatellite loci

	KYET	XSK	KLH	BEJ	CHE	HBH	TRKT
KYET		0.0153	0.02506	0.03535	0.02892	0.05988	0.10782
XSK	0.0928		0.03495	0.04505	0.04019	0.05932	0.09341
KLH	0.1641	0.223		0.02316	0.0388	0.03283	0.08905
BEJ	0.2129	0.2747	0.1868		0.01319	0.04169	0.09128
CHE	0.1607	0.2213	0.254	0.0942		0.05872	0.11049
HBH	0.4095	0.4049	0.2803	0.3325	0.4127		0.04656
TRKT	0.7483	0.602	0.6871	0.6909	0.7957	0.3227	

The AMOVA results illustrated that 94.34% of the total variance of nuclear genetics was attributed to the differences within populations (among individuals), and only 5.66% of the total variance was attributed to the differences among populations (Table), suggesting weak population genetic differentiation. The UPGMA dendrogram based on *F*_st_p_ values consisted of three main clusters. KYET, XSK, and KLH fell into the first cluster, which was converged with the second cluster of BEJ and CHE, and they were finally merged with the third cluster of HBH and TRKT (Figure 2b).

**Figure 3:**
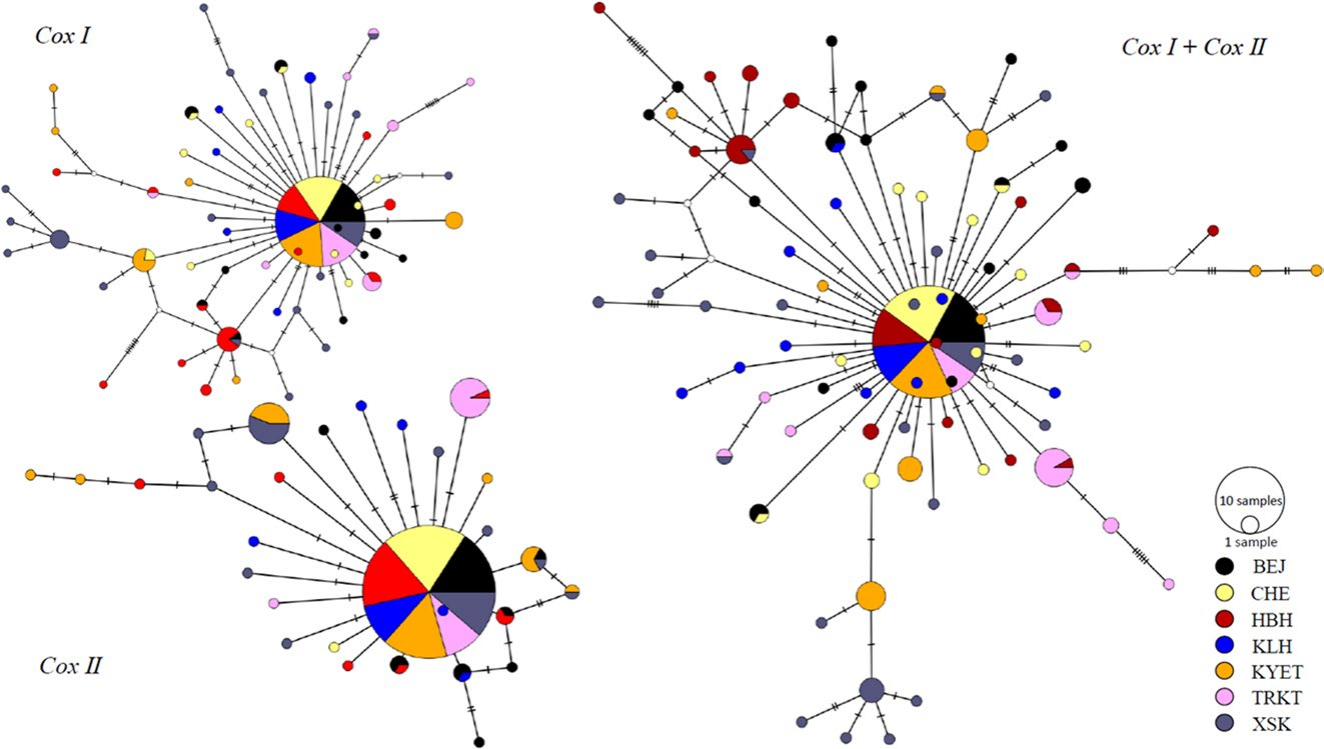
Haplotype networks based on three mitochondrial datasets. Variational size and percentage of colorized pies implied different numbers of haplotype shared by different populations. White circles represent intermediate haplotypes not observed. Networks were constructed in Popart v 1.7

As shown in Table 5, the low *G*_d_ values revealed by mtDNA haplotypes implied neither significant nor strong genetic structure among these populations. The shared haplotypes in the three networks suggested that some key cytoplasmic genetic information was communicated frequently across these populations, supporting the genetic structure to some extent (Figure 3).

**Table 5:** Genetic distance revealed from *Cox Ⅰ*/*Cox Ⅱ* （below diagonal，*Cox Ⅱ* in bold and italic）and *Cox Ⅰ* + *Cox Ⅱ* (above diagonal)

	KYET	XSK	KLH	BEJ	CHE	HBH	TRKT
KYET		0.00158	0.00097	0.00107	0.00084	0.00130	0.00125
XSK	0.00157		0.00137	0.00147	0.00123	0.00168	0.00164
	***0.00160***						
KLH	0.00078	0.00143		0.00074	0.00052	0.00099	0.00092
	***0.00130***	***0.00128***					
BEJ	0.00087	0.00151	0.00065		0.00062	0.00107	0.00103
	***0.00140***	***0.00140***	***0.00091***				
CHE	0.00078	0.00141	0.00057	0.00065		0.00086	0.00080
	***0.00094***	***0.00093***	***0.00044***	***0.00056***			
HBH	0.00135	0.00198	0.00116	0.00121	0.00117		0.00125
	***0.00121***	***0.00120***	***0.00072***	***0.00083***	***0.00036***		
TRKT	0.00104	0.00168	0.00082	0.00090	0.00083	0.00140	
	***0.00160***	***0.00159***	***0.00110***	***0.00123***	***0.00074***	***0.00099***	

**Figure 4:**
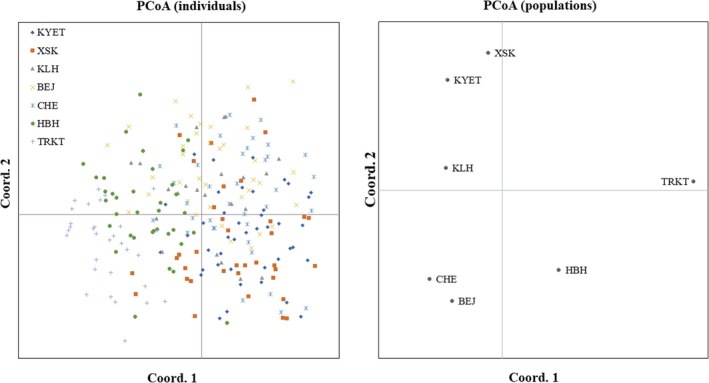
Principal coordinate analysis of pairwise distances between individuals and populations of *Phoxinus phoxinus ujmonensis*, visualized in GenAlEx 6.5

**Figure 5:**
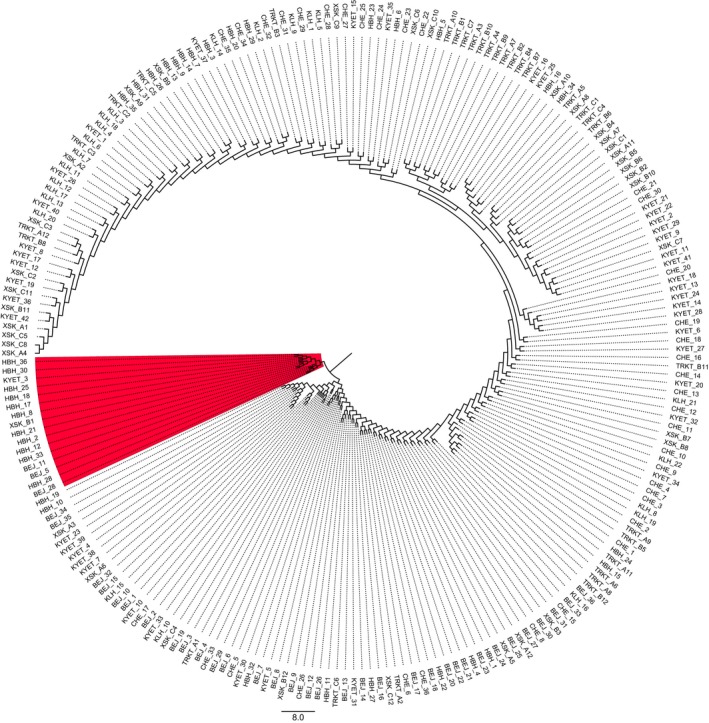
Maximum likelihood phylogenetic tree of 239 individuals based on the *Cox I* + *Cox II* dataset. The T92 + G model was determined and tree was constructed in Mega 6.0. The root clade (marked in red) consisted of 16 individuals only from populations inside or nearby the mainstream of the Irtysh River

Principal coordinate analysis based on pairwise individual genetic distance showed that most individuals were not distantly separated from each other. While, PCoA based on pairwise population genetic distance demonstrated that KYET, XSK, and KLH were in the lower left quadrant, BEJ and CHE in the upper left quadrant, HBH and TRKT in the lower and upper right quadrants, respectively (Figure 4). In addition, the maximum likelihood tree based on the T92 + G model showed that most individuals were randomly merged (Figure 5). However, the root clade (in red) consisted of 16 individuals only from populations inside or nearby the mainstream of the Irtysh River.

Admixture analysis suggested that these 239 individuals fell into three genetic clusters (*K* = 3), which was in accordance with the result of UPGMA dendrogram. Most individuals from KYET, XSK, and KLH fell into one cluster, while most individuals from BEJ and CHE and most individuals from HBH and TRKT were assigned to another two clusters, respectively (Figure 2c).

### 3.4 PCA and correlation analysis of environmental factors

**Figure 6:**
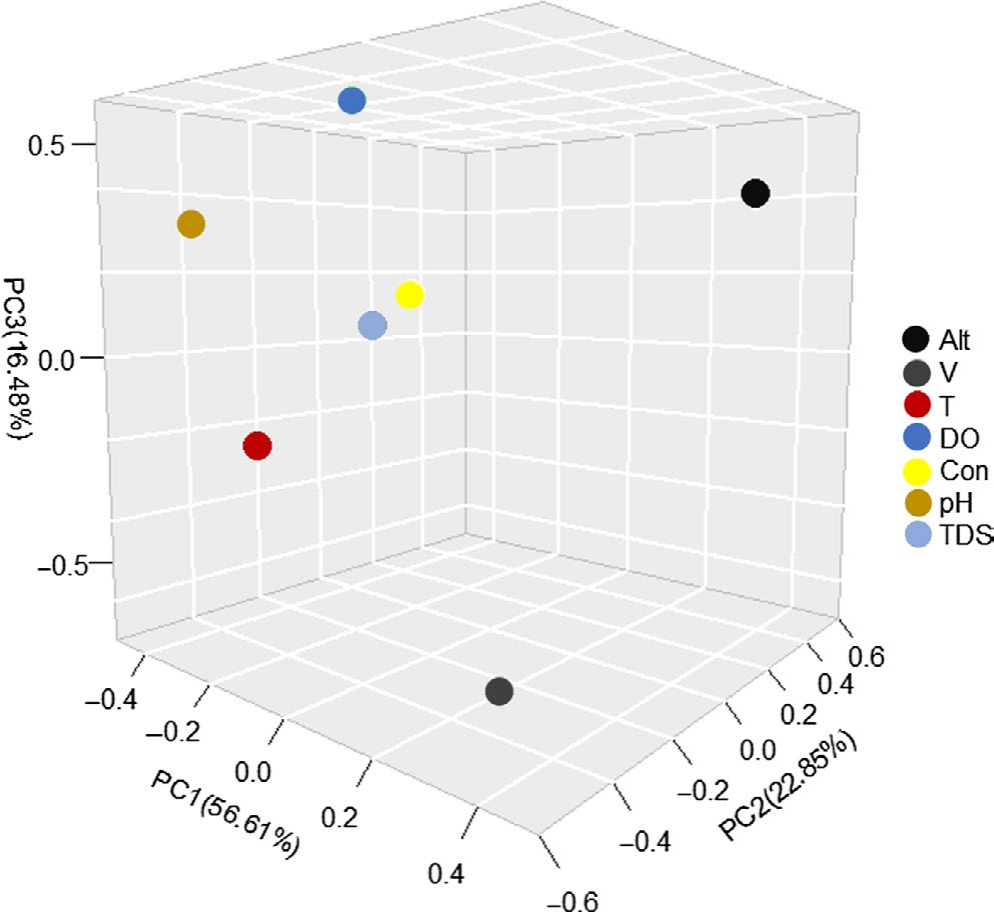
Principal component analysis of abiotic factors among the seven populations. The first three components, eigenvalues of which were greater than 1, accounted for 96.04% of the variance. PCA was calculated and visualized in R 3.5.3

**Figure 7:**
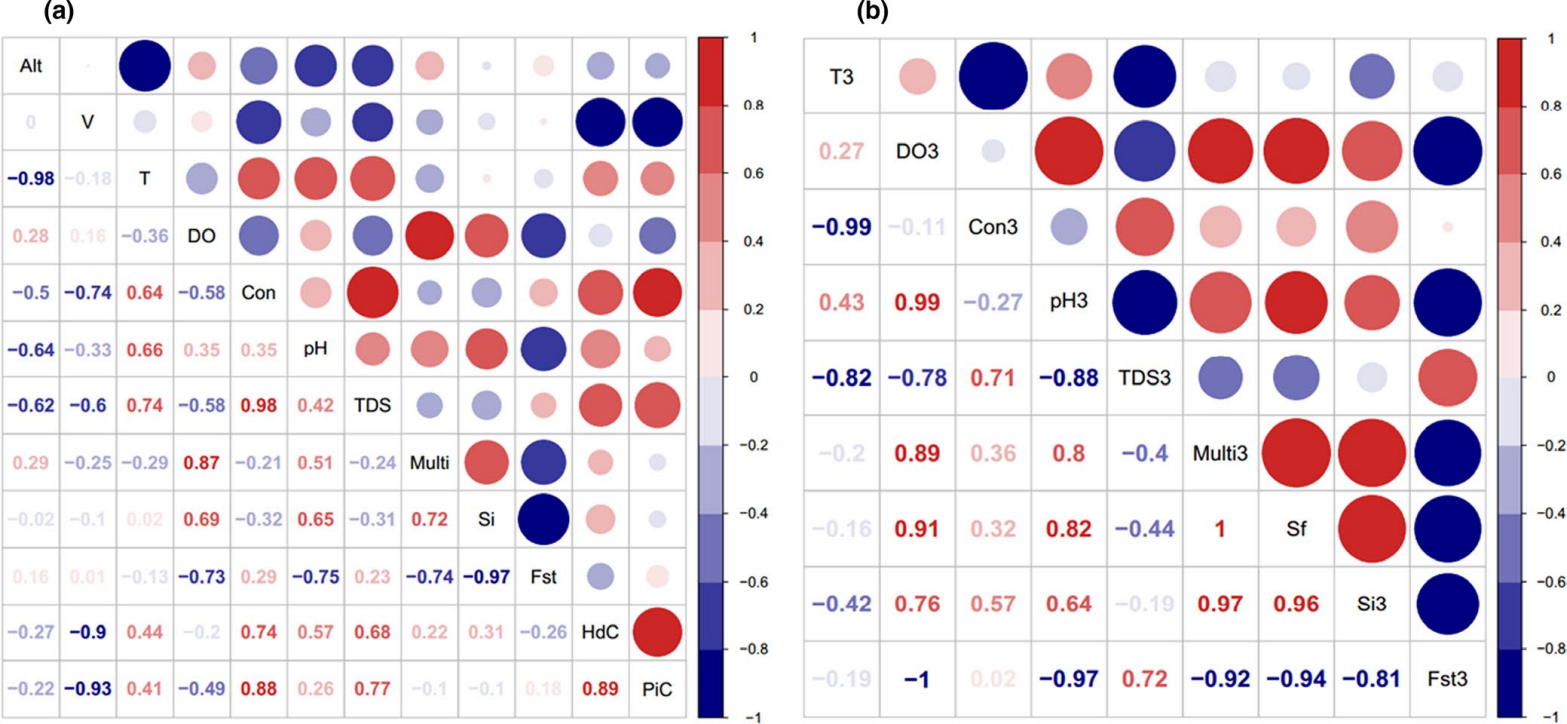
Correlation analysis between environmental factors and genetic diversity indexes, computed on both population level (a) and region level (b, the three regions were equal to the three clusters above, variables like “*T*” were marked with “3” to distinguish). Alt, altitude of sampling location; Con, conductivity of water; DO, concentration of dissolved oxygen; *F*_st_, population specific fixation index; HdC/PiC, haplotype/nucleotide diversity from *Cox Ⅰ* + *Cox Ⅱ*; Multi, mean values of plankton diversity indexes; pH, water pH; *S*_f_, Shannon's diversity index of fish species;* S_i_*, Shannon's information index from microsatellite loci; *T*, water temperature, TDS, total dissolved solids in water; *V*, flow velocity of water. Tests were conducted and visualized via “corrplot” package in R 3.5.3

The PCA results showed that the first three components accounted for 96.04% of the variance with their eigenvalues greater than 1 (Hayton, Allen, & Scarpello, 2004) (Figure 6). Notably, the loading values of *V* (flow velocity of water), DO, and pH were higher than other values with *V* of −0.635 in the third component, DO of −0.582 in the second component, and pH of −0.510 in the second component, respectively. The highest loading values in the first component were just −0.470 (TDS) followed by −0.453 (*T*) and −0.452 (Con). Haplotype diversity (HdC) and nucleotide diversity (PiC) were revealed by concatenate sequences of *Cox I* + *Cox II*, which presented most information of individual *Cox I* and *Cox II*. The correlation analysis showed that *S_i_* was positively related to two abiotic factors DO and pH, while *F*_st_ was negatively related to *S_i_* and these two factors. The HdC and PiC were positively related to Con and TDS, but negatively to *V* (Figure 7a). The average plankton diversity indexes of Mar, Sha, Sim, and Pie at each sampling site (data at TRKT site absent) were computed as Multi since these four indexes were highly positively correlated to each other. The correlation between *S_i_* and Multi was also positively high, while correlation between *F*_st_ and Multi was negatively high.

As both the UPGMA and admixture analysis suggested that these seven populations could be regarded as three clusters, we combined the allele data of individuals belonging to each cluster to calculate specific Shannon information index (Si_3_). We also computed the abiotic and biotic indexes in three regions corresponding to three clusters. In addition, we calculated the Shannon–Wiener's diversity indexes of fish species (*S*_f_) in the upper, middle, and lower regions (matched the three clusters). Visualized correlation analysis suggested that Si_3_ of the three regions was positively correlated to DO_3_ and pH_3_, as well as to Multi_3_ and *S*_f_ (Figure 7b).

**Figure 8:**
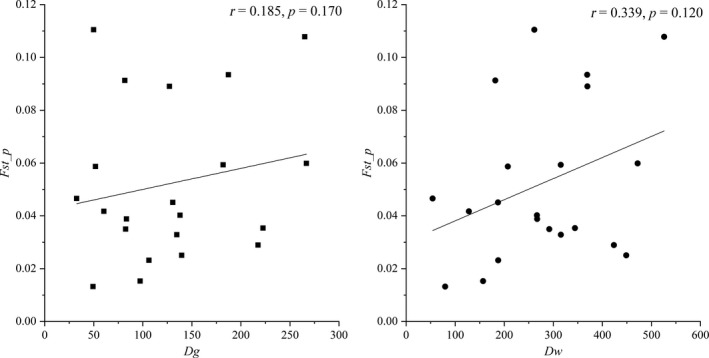
Mantel tests for detecting IBD patterns, matrixes of geographic (*D*_g_), and water (*D*_w_) distances against matrixes of *F*_st_p_. *D*_g_ and* D*_w_ were measured through Google Earth 7.1.2.2041. Tests were conducted via “vegan” package in R 3.5.3 and visualized in Origin 8.5

**Figure 9:**
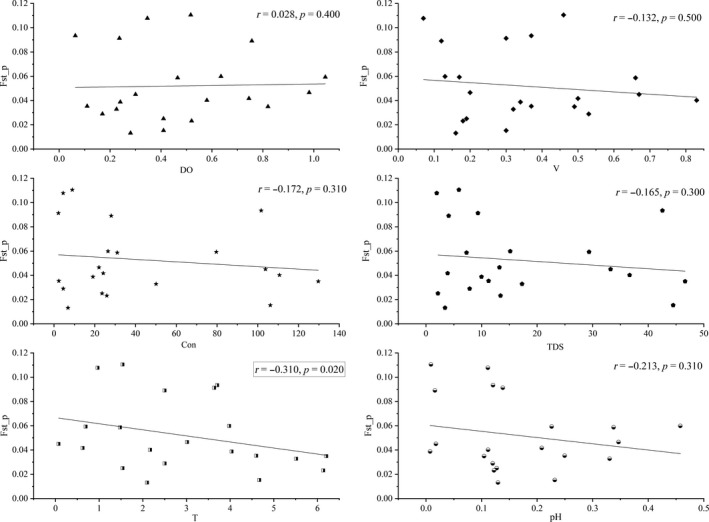
Mantel tests for detecting IBA patterns, matrixes of environmental variables against matrixes of *F*_st_p_. Con, conductivity of water; DO, concentration of dissolved oxygen; *T = *temperature of water; TDS, total dissolved solids in water; *V*, flow velocity of water. Tests were conducted via “vegan” package in R 3.5.3 and visualized in Origin 8.5

The results of Mantel test indicated that there were no IBD patterns on either geographic distance (*r* = 0.185, *p* = 0.170) or water distance (*r* = 0.339, *p* = 0.120; Figure 8). However, the slight but significant correlation (*r* = −0.310, *p* = 0.020) of the *T* matrix between *F*_st_p_ matrix might imply the potential IBA pattern, though correlations of *F*_st_p_ between other five factors were not significant (Figure 9).

## 4 DISCUSSION

### 4.1 Genetic diversity and structure

In this study, we found a high nDNA diversity in contrast with a moderate mtDNA diversity. Namely, (a) the mean *H*_o_ of these seven populations was as high as 0.791, and the mean *S_i_* was 1.976. Especially, the *S_i_* value, as the major index of the genetic diversity in this study, was found to be obviously higher than that in previous studies (Ji et al., 2014; Pandian et al., 2018; Wang et al., 2007). (b) Most *H*_d_ values inferred from *Cox I* or *Cox II* were close to the threshold value of 0.5 except XSK whose *H*_d_ value was as high as 0.852 at *Cox I*, and CHE whose *H*_d_ value was as low as 0.056 at *Cox II*. All the *P_i_* values were below the threshold value of 0.005 (Grant & Bowen, 1998). This contrast was consistent with the results of previous studies that employed these two types of markers (Guo et al., 2016; Kolleck, Yang, Zinner, & Roos, 2013; Pope, Estoup, & Moritz, 2010; Waits, Taberlet, Swenson, Sandegren, & Franzén, 2010), which could be attributed to the different inheritance patterns of these two types of markers. Nuclear genomes can be more stably inherited by the next generation, while cytoplasmic genomes could only be partially inherited from the previous generation (Galloway & Fenster, 1999; Jaj & Werren, 1995; Nigro, 1994). This suggests that the frequency of nuclear genotype declined much more slowly than that of cytoplasmic haplotype, resulting in high nDNA diversity but low mtDNA diversity in some species.

The pairwise genetic differentiation coefficients (*F*_st_p_) and genetic distances (*N*_d_) are main indexes of genetic structure. A large number of previous studies have provided the reference for the use of these two indexes (Hennink & Zeven, 1990; Nanninga & Manica, 2018; Rousset, 1997; Waldmann, Garcíagil, & Sillanpää, 2005). This study found that most *F*_st_p_ values were below the threshold value of 0.05, indicating no differentiation among these populations, except that two values of HBH and five values of TRKT were higher than 0.05 but lower than 0.15. This exception implied moderate differentiation among these populations (Wright, 1965). Like the *F*_st_p_ values, the *N*_d_ values also showed the same tendency of genetic distances among these seven populations.

It should be noted that the mean *H*_o_ values in the two populations located in the Haba River (0.821 in HBH and 0.802 in TRKT) were observed to be higher than others. The *S_i_* value in HBH (2.135) was also the highest. Additionally, the mean *F*_st_p_ and *N*_d_ values in HBH and TRKT were higher than those in the rest five populations with 0.050 and 0.368 in HBH, 0.098, and 0.705 in TRKT, respectively, which could be explained by the unique environment of the Haba River. For example, there existed several dams along the Haba River. The HBH and TRKT populations were located upper stream of the lowest and the second lowest dam ordinally, while streams and irrigation canals were scattered on both sides of the Haba River. Such an environment was favorable for the two populations' adaptation which allowed high genetic diversity and resulted in slight differentiation between these two populations and other five populations. Furthermore, the UPGMA dendrogram exhibited a slight but meaningful convergence; that is, these seven groups showed apparently regional aggregation in upper, middle, and lower reaches, respectively. This might reflect a trend that the genetic relationships among these seven populations developed along the river from upper stream to lower stream, since gene flow between nearby populations could occur more frequently. A similar trend was found in the population genetic structure of California Bocaccio and Atlantic Salmon (Matala, Gray, Gharrett, & Love, 2004; Primmer et al., 2010). Moreover, the NJ dendrograms of mitochondrial haplotypes presented a random pattern and no classification of phylogenetic haplogroups, as the haplotypes shared between geographically close populations were internally unrelated.

This weak but significant genetic structure could be explained by two hypotheses. First, the high nDNA and moderate mtDNA diversity together with moderate genetic differentiation among several populations might indicate that there existed extensive gene flow among these seven populations since these four tributaries were in a continuous water system, which could ensure high genotypic association of mainstream and tributaries. This might also explain the absence of IBD to some extent. The root clade in the ML tree which consisted of 16 individuals from populations inside or nearby the mainstream might also provide some interpretation for the association. The above results were generally concordant with the results of the genetic diversity and population structure analysis of *Atrina pectinanta* along the west coast of east Asian (An, Lee, & Mae, 2012; Xue, Wang, Zhang, & Liu, 2014). Second, human activities, such as construction of dams and fishing, might have had some impact on genetic exchange of fish like *P. phoxinus ujmonensis* widely distributed from upper stream to lower stream.

### 4.2 Influence of environment

It is well known that environment can shape both genetic and trait variation (Magalhaes, D'Agostino, Hohenlohe, & Maccoll, 2016; Robinson & Wilson, 1996). *P. phoxinus ujmonensis*' habit of preferring to inhabit in the cold and clean tributaries containing high dissolved oxygen may explain why the Shannon genetic information index (*S_i_*) was positively related to dissolved oxygen (DO). The previous study reported that dissolved oxygen availability might have great effect on the energy for fish locomotion, growth, and reproduction (1987). Generally speaking, the active locomotion could enhance frequent gene flow among fish populations, and efficient reproduction could ensure better inheritance of genetic information. The evidence supporting above findings could be found in the study of the population differentiation of the African cyprinid *Barbus neumayeri*. The aquatic dissolved oxygen was reported as a selective force limiting gene flow among populations and therefore played a key role in determining the genetic structure of populations (Robert, Merritt, Chapman, David, & Martinez, 2013). Genetic study in *Simulium tani* also showed that nucleotide diversity was strongly correlated to the levels of dissolved oxygen in the streams (Low, Adler, & Takaoka, 2014). The cold water fish *Cottus poecilopus* was also reported to be abundant in streams with high oxygen saturation and high abundance of macroinvertebrates (Baran et al., 2015). Another study of aquatic organisms found that among‐pond variation in community structure of freshwater zooplankton was partially explained by pH and dissolved oxygen (HolmesSingh, 2016). Similarly, the pH of water largely influenced the survival, growth, development, and reproduction of fish (Abbink et al., 2012; Ahmed, Donkor, Street, & Vasiljevic, 2013; Alavi & Cosson, 2005; Dziewulska & Domagała, 2013), which were also important for maintaining genetic information of species. For examples, the diversity of trout was previously reported to be low in lakes at low pH (Reimchen, 1992), and nine‐spined stickleback was abundant under alkaline conditions (MacColl, Nagar, & Roij, 2013). Thus, these findings should support the conclusion that genetic diversity is positively related to pH in this study, since the mean pH values among these seven populations ranged from 7.52 to 7.98.

However, genetic diversity and structure estimated by mitochondrial markers seemed to be influenced by abiotic factors like *V*, Con, and TDS. To be noted, *P*. *phoxinus ujmonensis* is a small‐body fish with weak swimming ability. And large females in fish usually represent sexually mature individuals who allocated more energy to reproduction rather than resistance against high flow velocity of water. Thus, we might speculate that the proportion of female *P*. *phoxinus ujmonensis* should be higher in the area with low velocity, especially in the breeding season. The HdC/PiC values in XSK, BEJ, and HBH populations were higher than those in the upper stream of the same tributary; that is, XSK was higher than KYET, BEJ was higher than CHE, and HBH was higher than TRKT. Considering the characteristics of maternal inheritance of mitochondrial genes, the negative correlation between *V* and HdC/PiC should be acceptable. The PCA of the dispersal tendency of pumpkinseed (*Lepomis gibbosus*) also showed the importance of water flow velocity (Ashenden, Rooke, & Fox, 2017). As a component of the cytoplasm and the energy factory of the cell, mitochondria directly and frequently respond to environmental stimuli. For example, it produces more energy to speed up swimming in response to some stimuli. Another variable phenotype directly related to cytoplasm in fish is the electroreception (Hopkins, 1976; Zakon & Meyer, 1983). Under certain conductivity in water, fish could rely on specific electroreceptors like jaw and lateral line to sense prey and predator (Bleckmann, 1986; Carr, Maler, & Sas, 2010; Janssen, 2010; King, Hu, & Long, 2018; Maciver, Sharabash, & Nelson, 2001). Thus, we could hypothesize that *P. phoxinus ujmonensis* populations with various plasticity of electroreception might be adapted to divergent conductivity conditions, resulting in divergence of haplotype and nucleotide diversity. Notably, the Pearson correlation coefficient between TDS and Con was as high as 0.98 among these populations, which might be attributed to the fact that along the north bank of the Irtysh River, the Altay mountains are rich in mineral resources. Obviously, dissolved solids with mineral ions attached contribute to maintain water conductivity. The early related study reported that the spawning migration of striped bass increased with the rise of TDS concentration below 300 ppm (Radtke & Turner, 2011), since certain fish preferred to reproduce at particular concentration of TDS (Chapman, Bailey, Canaria, Oris, & Klaine, 2010; Weberscannell, Duffy, & Weberscannell, 2007). Moreover, Chu, Ellis, and Kerckhove (2018) reported that TDS also acted as an important factor affecting fish assemblages. The above findings might support our results that HdC and PiC were statistically related to TDS. The presence of IBA, however, should be paid attention to. Though the correlation between differences of water temperature and *F*_st_p_ was weak, we need take further attention to the influence of habitat divergence aroused by temperature differences on genetic communication. The altitude drop of these four tributaries can be as high as 2,900 m. This should be the reason why the temperature difference was as high as 15.6°C between the XSK and KLH sampling sites in September 2015 due to the cold and variable mountain climate. (Table).

As for the biotic factors, the Shannon genetic information index (*S_i_*) was also highly positively related to both plankton diversity indexes (Mar, Sha, Sim, and Pie) and Shannon–Wiener's diversity indexes of fish species (*S*_f_). Food availability was reported to be an important factor influencing species richness, in turn, affecting genetic diversity (Gaston, 2000; Pope, Riginos, Ovenden, Keyse, & Blomberg, 2015). High diversity of plankton could guarantee enough food for fish like *P. phoxinus ujmonensis*, so that they might survive, grow, locomote, and reproduce well (Papiol, Cartes, & Fanelli, 2014). These lay a solid foundation for extensive gene flow. Previous studies also revealed that distribution and abundance of fish were positively associated with the abundance of their prey (Menezes et al., 2013; Nanjo, Kohno, Nakamura, Horinouchi, & Sano, 2014). Moreover, high diversity of fish species within the food web means more alternative species for predators like *L. lota* and *Sander lucioperca*. Therefore, as secondary consumers occupying the third trophic level, *P. phoxinus ujmonensis* can have more chances to survive rather than to be eliminated. Thus, genetic diversity could be maintained within stable populations. This is in accordance with the hypothesis that species diversity within communities might have potential positive effect on genetic diversity among populations via spatially varying selection (McCusker & Bentzen, 2010; Vavrek, 1998; Vellend & Geber, 2005). Specifically, the comprehensive study on species‐genetic diversity correlations (SGDCs) of four sympatric fish in the Garonne‐Dordogne river basin found that the α‐SGDCs for *P. phoxinus* were significant and positive, while the β‐SGDCs were also positive but weaker (Fourtune, Paz‐Vinas, Loot, Prunier, & Blanchet, 2016). Interestingly, plankton and fish species diversity are the vital components of trophic status and structure in a river system. Meanwhile, population diversity and structure of a certain species are largely affected by such trophic status and structure (Bart et al., 2015; Cebrian et al., 2009; Gordeeva, 2014; Herrera, Pozo, & Bazaga, 2011; Jeppesen, Peder Jensen, Søndergaard, Lauridsen, & Landkildehus, 2010; Utne‐Palm et al., 2010).

Nonetheless, one might note the discrepancy that *S_i_* (nuclear diversity) was positively related to DO but HdC/PiC (cytoplasmic diversity) were positively related to TDS since DO and TDS were always negatively related. We thought that this should also be due to the different inheritance patterns of these two types of markers mentioned above and the unique environment in this watershed. The four tributaries wind down the hillside on the north side of the mainstream. The TDS, to which HdC/PiC were positively related, were undoubtedly higher in the XSK, BEJ, and HBH populations since they were located in the lower reaches of the three tributaries, while the DO seemed to be higher in upper reaches except for the HBH population. Nevertheless, the Pearson correlation coefficient for *S_i_* and DO (0.69) was lower than that for *S_i_* and Multi (0.72), and they were both lower than that for DO and Multi (0.87). As the Multi represented the abundance of food for *P. phoxinus ujmonensis*, we might hypothesize that food availability has more influence on the genetic diversity of this species.

## 5 CONCLUSION

In this study, we developed and characterized 12 highly polymorphic microsatellite loci. Population analysis revealed high level of genetic diversity in local populations of *P. phoxinus ujmonensis* along the Irtysh River, which might be attributed to high genotypic association ensured by the continuous water system. However, there was moderate genetic differentiation among several populations, which could partially be due to the habitat fragmentation. The slight but meaningful differentiation of TRKT population against other populations might imply that human activities are unneglectable factors that influence genetic structure of fish. There was no obvious evidence supporting isolation by distance. However, we found the potential evidence of isolation by adaptation as well as the highly positive correlation between genetic diversity and abiotic or biotic factors. This might also suggest that there exist gene flow along distances, but different populations might be in the process of adapting to divergent environments due to habitat fragmentation. Finally, as *P. phoxinus ujmonensis* occupies an important niche in the food web, our results will provide reference for the study of population genetics of economic or endangered fish in the same catchment. The relationship of genetic information of various fish species and the detailed interaction between environmental factors and genetic characteristics remain to be further investigated.

## CONFLICT OF INTEREST

The authors declare no conflicts of interest.

## AUTHOR CONTRIBUTIONS

Peng Xie and Xu‐Fa Ma designed the research. Peng Xie, Guang Zhao and Jian‐Gong Niu performed the sampling work related to fish individuals. Jun Wang and Qiong Zhou performed the sampling and data analysis work related to plankton and abiotic factors involved. Peng Xie conducted the statistical analysis of genetic data and environmental data. Peng Xie drafted the manuscript. Xu‐Fa Ma and Yan Guo revised the manuscript. All authors reviewed the manuscript.

## Data Availability

Microsatellite genotypes for seven populations were in Table. (Tables) were in the Supporting Information.
